# Conventional Frenectomy With Topical Ozonated Oil Application

**DOI:** 10.7759/cureus.55522

**Published:** 2024-03-04

**Authors:** Shrishty Priya, Amit Reche, Prasanna R Sonar

**Affiliations:** 1 Dentistry, Sharad Pawar Dental College and Hospital, Datta Meghe Institute of Higher Education & Research (Deemed to be University), Wardha, IND; 2 Public Health Dentistry, Sharad Pawar Dental College and Hospital, Datta Meghe Institute of Higher Education & Research (Deemed to be University), Wardha, IND; 3 Oral Medicine and Radiology, Sharad Pawar Dental College and Hospital, Datta Meghe Institute of Higher Education & Research (Deemed to be University), Wardha, IND

**Keywords:** conventional scalpel technique, gingival recession, midline space closure, midline diastema, labial frenectomy

## Abstract

The frenum is a mucous membrane fold that connects the lip and cheek to the gingiva, periosteum, and alveolar mucosa. When the frena are linked too closely to the gingival border, there may be issues with plaque removal, and the overall gingiva may be affected. In addition, the maxillary frenum may provide aesthetic difficulties or interfere with the aesthetic outcome in cases of midline diastema, which may result in a recurrence after treatment. A labial frenectomy, a frequently performed surgical operation in the specialty of dentistry, is used to address such an abnormal frenum. This article describes a case study of a maxillary labial frenectomy using a traditional scalpel approach and topical application of ozonated olive oil.

## Introduction

The frenum connects the lip and cheek to the alveolar mucosa, gingiva, and the periosteum beneath it. When the frena are linked too closely to the gingival border, because of problems with plaque clearance, the health of the gingiva may be affected and can result in gingival recession. Furthermore, in patients with midline diastema, the frenum may create aesthetic challenges, which could lead to a recurrence after orthodontic treatment. Among the most frequent issues that patients bring to a dental clinic are midline diastema and spacing. The main issue with these cases is their unappealing appearance. As seen in this case, aberrant frenal attachment is the main cause. In order to have an ongoing course of treatment, the primary cause must be eliminated. A frenectomy was carried out in this instance to fix the aberrant frenal association. A frequent surgical operation in the field of dentistry is the labial frenectomy, which is used to handle such an aberrant frenum [1].

Focusing on the frenum has become significant since it is an aetiological variable contributing to the maintenance of a midline diastema [2]. According to the degree of fiber attachment, the frenum is divided into four categories: mucosal, gingival, papillary, and papilla piercing [3]. An ectolabial band connects the tubercle of the upper lip and the palatine papilla, which eventually results in the maxillary labial frenum. The central incisors erupt far apart, but no bone is deposited beneath the frenum. The outcome is an aberrant frenum attachment and a V-shaped bone fissure between the central incisors [4].

Treatment options for the aberrant frenulum include frenectomy or frenotomy. A frenectomy is the removal of the frenulum in its entirety, including the fibrous connection that keeps it attached to the periosteum and alveolar bone. Without removing the collagen fibers that are firmly linked, a frenotomy is the repositioning or more superficial removal of the frenal attachment [5,6]. An improperly executed surgery may expose portions of the root surface, especially during a frenectomy. The exposure is known as bone dehiscence if the root lacks alveolar bone covering from the marginal toward the apical area. A fenestration is an exposed root with a minimally undamaged strip of bone.

This article describes a frenectomy case that was performed using a standard scalpel approach and topical ozonated oil administration. The main goal of this technique was to correct the labial frenum's excessive attachment, which was a contributing factor to the noticeable maxillary midline diastema.

## Case presentation

**Figure 1:**
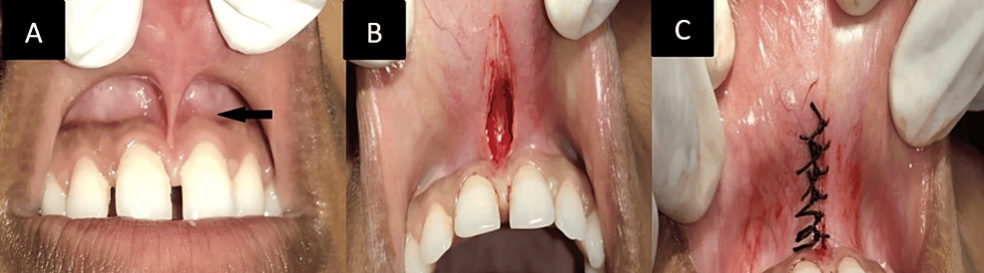
A: high frenum attachment; B: V-shaped incision on the undersurface of the frenal attachment; C: suturing

A 25-year-old male patient's main concern upon visiting the dental hospital was teeth spacing. There was no relevant systemic and dental history. On examination, the abnormal high positioning of the frenum was identified through a blanch test. The abnormal high positioning of the frenum is shown in Figure 1A, marked by its width and insufficient zone of the attached gingival along the midline. A conventional surgical procedure was planned, and written consent was acquired before the intervention. A hemostat, a no. 15 scalpel blade, a gauze sponge, 4-0 black silk sutures, suture pliers, and scissors were all included in the surgical toolbox. The assessment and analysis of the frenum and its surrounding area were conducted. The region was anesthetized using a 2% lignocaine local infiltrate with 1:80,000 adrenaline. The hemostat was used to secure the frenum, followed by making a V-shaped incision on the undersurface of the frenal attachment, as illustrated in Figure 1B. Adequate undermining of the surrounding tissue was executed to ensure proper mobilization of flaps and minimize damage to underlying structures. The two flaps were positioned in such a way that their apices faced each other. Subsequently, they were sutured to the defect on the opposite side of the other flap using interrupted braided silk sutures, as shown in Figure 1C. Silk sutures were used because they are easy to handle, provide sufficient strength to tissue approximation, and cause less irritation to the patient [7].

**Figure 2:**
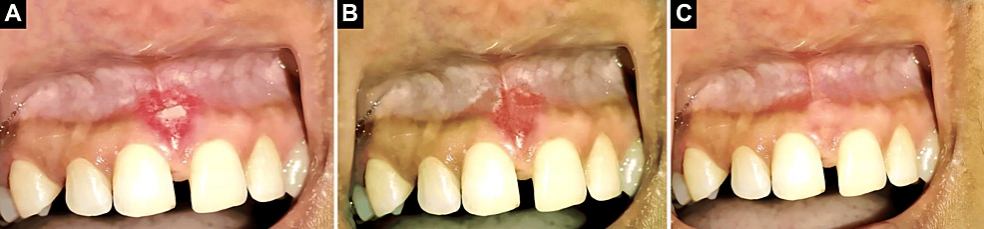
A: Fourth day follow-up visit; B: fifth day follow-up visit; C: 14th day follow-up visit.

Following the procedure, the patient was only advised topical ozonated oil application (three times daily for at least five minutes). A review of the literature suggested that topical application of ozonated oil promotes wound healing [8], so we decided to choose ozonated oil for topical application. On the fourth day of follow-up, the sutures were removed. The frenum had successfully been repositioned to the apical position, and the healing process was satisfactory. The use of ozonated oil contributed to facilitating satisfactory healing, as presented in Figure 2A, Figure 2B, and Figure 2C.

## Discussion

Scalpel conventional frenectomy is the first and oldest method of frenectomy presented. The necessity of receiving dental care has increased as a result of aesthetic considerations. Concern over the frenum has become essential since an aberrant frenum is a cause for the recurrence of a midline diastema. When the frenal attachment is too close to the gingival margin, it results in gingival recession. Archer's conventional frenectomy approach is a complex procedure that results in interdental papilla loss and scarring [9,10]. Conservative methods include the free gingival graft, Z-plasty, and Edward's frenectomy [11]. Several modifications have been made to address the problems brought on by an abnormal labial frenum. However, the majority of treatments do not take aesthetics or the zone of attached gingiva into account; instead, surgical frenectomy techniques, such as the conventional (classical) technique, are used [2]. The results of frenectomy with a bilateral pedicle flap and Miller's unilateral pedicle flap approach are reported [12,13]. The pedicle flap frenectomy approach yields good cosmetic outcomes, color matching, increased gingiva attachment, and no anesthetic scar formation because primary intention healing occurs. The ideal patient for Z-plasty surgery was found to have a thick, wide, hypertrophic frenum positioned low and coupled to a short vestibule and an inter-incisor diastema [14].

With the least amount of trauma to the surrounding tissues, the conventional technique provided the benefits of precision and control over tissue removal. In addition, the patient recovered from the procedure quickly and with little discomfort. There are a few risks associated with surgical frenum removal, including intraoperative hemorrhage, bleeding, infection, scarring, damage to the periodontal ligament, and patient anxiety, among others. At the moment, numerous other frenectomy procedures have been developed, including electrocautery, diode, and Nd: Yag laser frenectomy in combination with other techniques [15-18]. Despite their advancements, these methods come with various drawbacks, such as high costs, post-operative pain, and edema. Technique-sensitive procedures that require a clean, precise incision for appropriate tissue approximation after surgery, such as V-Y and Zplasty, are challenging to execute with lasers.

It has been demonstrated that topical administration of ozonated oil is effective at gingival surgery sites [8]. After topical ozonated oil administration, epithelial healing was satisfactory in the case, which was presented. Scarring or other issues related to the current case did not occur.

## Conclusions

A relatively common issue in the general population is high frenum attachment. Moreover, maintaining proper dental hygiene is hampered by high frenum. Simultaneous orthodontic therapy and frenectomy can more reliably result in the closing midline diastema with a pronounced frenum. This case illustrated the effectiveness of the traditional scalpel method combined with topical ozonated oil treatment in reaching the intended surgical result.
